# 620. Pharmacokinetic-Pharmacodynamic Analysis of Oral Vancomycin and Gut Microbiome Changes in Healthy Volunteers: an Exploratory Study

**DOI:** 10.1093/ofid/ofac492.672

**Published:** 2022-12-15

**Authors:** Jinhee Jo, Jacob McPherson, Anne J Gonzales-Luna, Chris Lancaster, Kevin W Garey

**Affiliations:** University of Houston College of Pharmacy, Houston, Texas; University of Houston College of Pharmacy, Houston, Texas; University of Houston, Houston, Texas; University of Houston, Houston, Texas; University of Houston College of Pharmacy, Houston, Texas

## Abstract

**Background:**

Oral vancomycin causes profound changes to the gut microbiome due to high intra-colonic vancomycin concentrations. However, pharmacokinetics of oral vancomycin causing pharmacodynamic changes have not been explored, especially during the early dosing period. The purpose of this study was to investigate fecal vancomycin concentrations in healthy individuals in relation to gut microbiome diversity changes.

**Methods:**

Healthy subjects 18-45 years with no antibiotic use for at least 28 days were given oral vancomycin 125 mg was given every 6 hours for 10 days. Stool samples were collected at baseline and during antibiotic therapy. Vancomycin concentrations were obtained through high-performance liquid chromatography (HPLC) assay. For this early pharmacokinetics analysis, stool samples from day 0 (baseline) to day 4 were included. Shotgun metagenomics sequencing was used for microbiome analysis. Descriptive analysis was performed to identify gut-microbiome phyla changes in correlation with detectable oral vancomycin fecal concentrations.

**Results:**

A total of 6 healthy volunteers aged 32±5 years (Male: 100%; Caucasian: 50%; mean BMI: 26.8±4.5 kg/m^2^) were included. In the early dosing period (day 0-4), the mean fecal vancomycin concentrations increased daily with the highest concentration of 1,586 μg/g of stool occurring on day 4. Three of 9 subjects (50%) had undetectable oral vancomycin levels one days 1 and 2 of dosing. Within 24-48 hour of detectable vancomycin levels, subject-specific changes of gut microbiome phylum-level proportions were observable. Overall, an increase in Actinobacteria and Proteobacteria phyla and decrease in Firmicute phylum was observed within 24 hours that vancomycin was detected in the feces.

**Conclusion:**

High concentrations of vancomycin are achieved in the stool by day four of dosing for all subjects; however, low concentrations are observed early in the dosing period for some subjects. Proportional, subject specific differences in gut microbiome phyla were observed within 24 hours of detectable vancomycin levels in the feces.

**Disclosures:**

**Kevin W. Garey, PharmD, MS**, Acurx Pharmaceuticals: Grant/Research Support|Paratek Pharmaceuticals: Grant/Research Support|Seres Therapeutics: Grant/Research Support|Summit Pharmaceuticals: Grant/Research Support.

